# Clinical in‐vivo dosimetry for total skin electron therapy using gafchromic™ EBT4 film

**DOI:** 10.1002/acm2.70774

**Published:** 2026-08-28

**Authors:** Zhexuan Zhang, Lan Lu, Gregory M. M Videtic, Cherian Sheen, Ping Xia, Peng Qi

**Affiliations:** ^1^ Department of Radiation Oncology Cleveland Clinic Foundation Cleveland Ohio USA

**Keywords:** film dosimetry, in vivo dosimetry, TLD, total skin electron therapy

## Abstract

**Background:**

Total skin electron therapy (TSET) is a standard treatment for cutaneous T‐cell lymphoma. Due to the complex patient positioning and irregular body contours, robust in‐vivo dosimetry (IVD) is essential to verify dose uniformity. While thermoluminescent dosimeters (TLDs) are traditional standard, their utility is hindered by labor‐intensive, manual processing.

**Purposes:**

This study evaluates the clinical implementation of a Gafchromic™ EBT4 film‐based IVD system integrated with a bespoke, open‐source analysis platform for automated batch‐processing. To address the inherent orientation dependence and loss of film orientation frequently encountered when preparing small‐format IVD films with a manual paper cutter, we developed an orthogonal dual‐scan protocol. By averaging pixel values from two perpendicular scans for both calibration and clinical measurement, this protocol effectively mitigates orientation‐dependent uncertainties and ensures dosimetric robustness.

**Methods:**

The film‐based IVD system was clinically assessed in five TSET treatments and compared to six treatments utilizing TLD‐based IVD. Dosimetric accuracy was evaluated by comparing normalized fractional doses to the prescription. Statistical analysis was performed using the Mann‐Whitney U test with the Benjamini‐Hochberg procedure to control the false discovery rate across 20 anatomical sites. Workflow efficiency was quantified by the total time required for data readout and analysis per patient.

**Results:**

The EBT4 film‐based IVD demonstrated dosimetric accuracy comparable to the TLDs, with mean fractional doses of 102.7% ± 9.6% and 100.4% ± 9.7%, respectively (*p* = 0.25). Clinical implementation of the film‐based approach significantly enhanced efficiency, reducing the total processing time from approximately 50 min to 15 min per treatment.

**Conclusions:**

The proposed EBT4 film‐based IVD system, supported by open‐source software and an orthogonal dual‐scan protocol, offers a robust, cost‐effective, and time‐efficient alternative to traditional TLDs. This approach streamlines the clinical workflow without compromising dosimetric accuracy, making it a viable solution for TSET and broader radiotherapy IVD applications.

## 1 INTRODUCTION

Total Skin Electron Therapy (TSET) is a well‐established and effective treatment modality for patients with cutaneous T‐cell lymphoma, otherwise known clinically as mycosis fungoides (MF).^1^, ^2^, ^3^, ^4^ The primary dosimetric objective of TSET is to deliver a uniform dose across the entire skin surface while minimizing exposure to deeper tissues. American Association of Physicists in Medicine (AAPM) Task Group 30 recommends maintaining dose uniformity within ± 8% in the vertical direction and ± 4% in the horizontal direction.^5^

TSET is commonly delivered using the dual‐field Stanford technique, in which the patient stands and rotates through six treatment positions. At each position, two angled electron beams delivered at an extended source‐to‐surface distance (SSD).^5^, ^6^ While traditional prescription dose ranged from 3,000 to 3,600 cGy, clinical management of MF has recently shifted toward low‐dose regime, typically 1,200 cGy. Clinical evidence suggests that low‐dose TSET achieves response rates comparable to traditional protocols while significantly reducing treatment‐related toxicities.^7^, ^8^, ^9^

In clinical practice, achieving the required dose uniformity is technically challenging due to patient positioning variability, complex surface anatomy, and self‐shielding in concave regions.^10^ Consequently, in‐vivo dosimetry (IVD) has become an essential for TSET to verify surface dose delivery. Among the existing IVD systems, thermoluminescent dosimeters (TLDs) have been most widely used.^10^ However, TLD‐based IVD for TSET is inherently labor‐intensive, requiring meticulous manual handling and prolonged readout procedures. As a result, many centers have adopted film‐based IVD using Gafchromic™ EBT2 or EBT3 films.^11^, ^12^, ^13^, ^14^, ^15^

In late 2023, we started initiated a transition from TLD‐100 dosimeters (Quantaflux LLC, Dayton, Ohio) to Gafchromic™ EBT4 films (Asland Inc., Wilmington, Delaware) for TSET IVD. The decision to adopt EBT4 over previous EBT3 films was motivated by two major factors: the anticipated discontinuation of EBT3 and recent literature demonstrating the enhanced performance of EBT4 film, including minimal energy dependence and improved signal‐to‐nose‐ratio.^16^

Additionally, unlike many prior studies that mostly utilized commercial software, we developed a bespoke, open‐source analysis platform in Python to standardize film calibration and automate dose extraction. To further enhance dosimetric robustness, an orthogonal dual‐scan protocol was implemented for both calibration and IVD measurement. This strategy was specifically adopted to mitigate the uncertainties associated with the loss of film orientations occurred when simultaneously cutting multiple small‐format IVD films using a manual paper cutter.

In this study, we performed clinical evaluation of this EBT4‐based IVD for TSET treatments and provide a rigorous framework for film preparation and handling, orthogonal dual‐scan strategy in calibration and IVD measurement and streamline dose analysis. To our knowledge, this work constitutes the first comprehensive report to establish clinical protocols for Gafchromic™ EBT4 film‐based IVD for TSET using a bespoke, open‐source platform.

## 2 METHODS AND MATERIALS

### 2.1 TSET treatment

Total skin electron therapy is performed at our institute using the dual‐field Stanford technique. Patient positioning is facilitated using a dedicated total body irradiation (TBI) stand (Radiation Products Design Inc., Albertville, MN), which features a vertically adjustable platform to accommodate varying patient heights. The platform includes angular reference markings at 60° increments to ensure reproducible patient rotation. Additionally, the stand is equipped with two adjustable hand grips that can be repositioned vertically and laterally to enhance patient stability and maintain consistent posture.

Six treatment positions are used: anterior‐posterior (AP), right anterior oblique (RAO), right posterior oblique (RPO), posterior‐anterior (PA), left posterior oblique (LPO), and left anterior oblique (LAO). These positions correspond to platform angular markings of 0°, 60°, 120°, 180°, 240°, and 300°, respectively.

For each treatment position, the SSD is set to 350 cm, measured to the most protruding anatomical point of the patient. This distance is verified by a medical physicist using a floor reference line located 250 cm from the gantry crosshair projection at 0°. At each position, the patient is irradiated with two divergent 6 MeV electron beams angled 18° above and below the horizontal axis (the “dual‐field” approach). Beams are delivered in high dose (HD) rate mode (2,500 monitor units (MUs) per minute) on a TrueBeam linear accelerator (linac; Varian Medical Systems, Palo Alto, CA). A customized 6‐mm thick acrylic spoiler plate is inserted into an electron cone mount to broaden the beam and increase surface dose. Because the spoiler is not electronically interlocked with the treatment mode, a manual override is required and verified by the physicist prior to each delivery.

The standard prescription dose of 1,200 cGy is delivered over 12 days (4 days per week) within a three‐week period. One fraction (200 cGy) is split across two days: the first day covers the AP, LPO, and RPO positions, and the following day covers the PA, RAO, and LAO positions. We had an accelerated case in which the regime was compressed to 8 days over two weeks.

### 2.2 TLD‐based IVD

#### 2.2.1 TLD calibration

For reference calibration, four TLD‐100 capsules were positioned within a custom‐milled recess in an acrylic phantom. The depth of the recess was specifically machined to match the diameter of the TLD capsules, ensuring a flush and reproducible geometry. A Solid Water buildup layer was placed on top of the acrylic phantom; the combined thickness of the buildup layer and the radius of the TLD capsules was selected to match the depth of maximum dose (d_max_) on the central‐axis percentage depth dose (PDD) curve.

A 20 MeV electron was selected for TLD calibration rather than the clinical 6 MeV beam. This choice was made because the 6 MeV PDD curve exhibits a steep dose gradient near the nominal d_max_. Utilizing the higher energy mitigates dosimetric uncertainties related to the setup.

The reference TLDs were irradiated under reference conditions: a 15 × 15 cm^2^ cone, 100 cm SSD, and a 200 MU delivery. Each TLD was processed using a Harshaw Model 3500 TLD reader (Thermo Fisher Scientific Inc., Waltham, MA). The dose calibration factor was calculated as the ratio of the delivered dose of 200 cGy to the arithmetic mean of the charge readings from the four reference TLDs.

#### 2.2.2 Clinical TLD IVD

**FIGURE 1:**
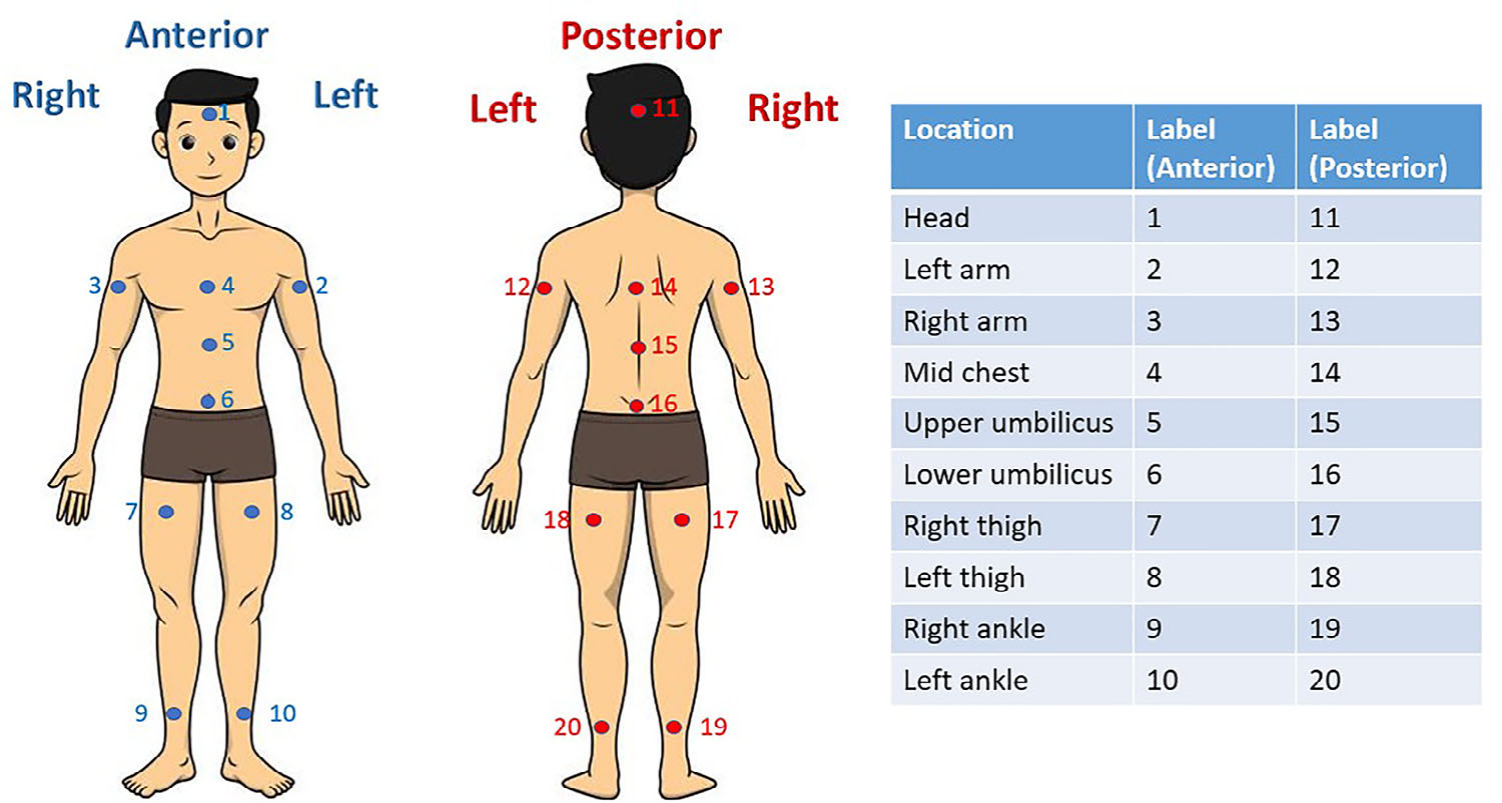
Anatomical locations of in‐vivo dosimeters for total skin electron therapy. Twenty specific measurement points are distributed across the patient's anterior and posterior skin surface to assess the delivered dose during the first treatment fraction.

For TSET IVD, a total of 40 TLD‐100 capsules were utilized, organized into 20 dosimeter packets (two capsules per packet). Each packet was uniquely numbered and secured to its designated location (see Figure 1) on the patient's skin using medical‐grade tape. The TLD placement was repeated for the first two treatment sessions to capture the fractional dose from all six treatment positions.

To ensure signal stability, the TLDs were processed approximately 24 h after the completion of second treatment day. For each anatomical site, the dose was determined by calculating the arithmetic mean of charge readings from the two paired TLDs and applying the previously established calibration factor.

### 2.3 Film‐based IVD

#### 2.3.1 Film preparation

For calibration purposes, Gafchromic™ EBT4 films were cut into approximately 4 × 4 cm^2^ squares using a guillotine‐style paper cutter. To ensure the consistence of the film orientation, a corner was notched on each calibration segment. These films were immediately sealed in light‐tight envelopes to prevent exposure to ambient and ultraviolet light prior to irradiation.

For clinical IVD, films from the same manufacturing lot as the calibration set were cut into small segments, typically 1.5 × 1.5 cm^2^ or smaller. To maximize efficiency, multiple segments were often cut simultaneously using a manual paper guillotine.

To create the dosimeter packets, two film segments were paired and wrapped in the manufacture‐provided protective paper originally used to separate film sheets. Prior to use, all prepared packets were stored in a light‐tight envelope.

#### 2.3.2 Film calibration and irradiation

For calibration or the establishment of the dose‐response relationship, a set of 13 calibration films were irradiated. To mitigate the influence of electron contamination form the linac head, each film was positioned at a depth of 10 cm with a Solid Water phantom with 10 cm of back‐scatter material.

Calibration was performed using a 6 MV photon beam with a 10 × 10 cm^2^ field size and 100 cm SSD. The 13 dose levels ranged from 0 to 800 cGy with a higher density of data points around 200 cGy to improve the fit in the expected clinical dose range. The MUs required for each dose level were calculated based on the current monthly quality assurance (QA) output factors and commissioning PDD data.

#### 2.3.3 Clinical film IVD

For clinical IVD, 20 film dosimeter packets were uniquely labeled and secured to the designated anatomical sites on the patient's skin using medical‐grade tapes. Consistent with the TLD protocol, these packets were placed prior to the start of treatment and removed after irradiation for each of the first two treatment days. When not in use, the film packets were stored in light‐tight envelopes.

#### 2.3.4 Film scanning and orthogonal dual‐scan protocol

To ensure consistency and film signal stabilization, all films were scanned for approximately 24 h after the irradiation on the second treatment day. To minimize lateral response artifacts (LRA), all films (calibration and IVD) were positioned within a 5 × 10 cm^2^ region at the center of the scanner bed. Films were scanned on an Epson Precision 4900 flatbed scanner (Epson Corporation, Nagano, Japan) with the following parameters: Professional mode, 48‐bit Color, Film (with Film Area Guide), Positive Film, 150 dpi resolution, and Color Correction disabled. Each scan was then saved in TIFF format.

In high‐throughput practice of preparing small IVD films, the original film orientation is frequently lost during cutting and handling process, and thus traditional method of preserving orientation, such as notching a corner, becomes impractical. This loss of orientation in combined with the film's inherent dichroic properties can introduce significant dosimetric errors if the traditional single‐scan approach is used.

This challenge served as the primary motivation for our orthogonal dual‐scan protocol. By averaging the pixel values from two perpendicular scans, the system mathematically compensates for unknown film orientations, ensuring dosimetric robustness and accuracy regardless of film orientation.

#### 2.3.5 Film analysis with open‐source software

In the absence of accessible proprietary commercial software tailored for this film‐based IVD, a bespoken analysis platform was developed in house using Python (version 3.11) and the source code has been made openly available via a GitHub repository (https://github.com/Applizium/Film‐Dosimetry‐App). The software architecture—including the dose‐response curve‐fitting algorithms, the batch‐processing workflow, and the graphical user interface (GUI)—was conceptualized and defined by the authors to meet the specific clinical requirements of TSET IVD. The translation of these specifications into functional Python code was facilitated by a generative AI coding assistant (Gemini 2.5 Pro, Google LLC). All AI‐generated code was rigorously audited, verified for dosimetric accuracy, and integrated by the authors to ensure compliance with institutional and clinical standards.

For the establishment of dose‐response calibration curve, the 13 calibration films were digitized using the orthogonal dual‐scan protocol. For each calibration dose level, mean pixel values (MPV) were extracted from a central 2 × 2 cm^2^ region of interest (ROI) in both orientations (MPV_horizontal_ and MPV_vertical_) from the red, green, and blue channels simultaneously.

The orientation‐independent average PV (PV_ave_) was calculated as the arithmetic mean of MPV_horizontal_ and MPV_vertical_. The relationship between the delivered dose and PV_ave_ was modeled using a rational function:

$$D=a+\frac{b}{PV_{ave}-c}$$

where *a*, *b*, and *c* are the fit parameters. The goodness‐of‐fit was verified by calculating the coefficient of determination (R^2^).

For clinical IVD, the same dual‐scan protocol was applied to all patient films. The orientation‐independent response was extracted from 1 × 1 cm^2^ ROI at the center of each IVD film. This PV_avg_ was then used through the analysis platform to automatically calculate delivered dose based on the established dose‐response calibration curve.

### 2.4 Comparison of TLD‐ and film‐based IVD

#### 2.4.1 Dose variability

To evaluate the consistency of the TLD‐ and film‐based IVD systems, two types of dose variability were assessed:

1. interpatient variability: defined as the dose variation between individual patients within each film and TLD cohort.
2. intrapatient variability: defined as the dose variation across the 20 different anatomical sites on a single patient, analyzed separately for each cohort.

Because of variations in prescribed fractional doses, the measured fractional doses were normalized to patient's specific prescribed fractional dose. Results are reported as a percentage of the prescribed fractional dose.

Statistical comparisons of dose variability between the two cohorts were performed using the Mann‐Whitney U test. To mitigate the risk of Type I errors inherent in simultaneous comparison across 20 anatomical sites, the Benjamini‐Hochberg procedure was applied. This correction adjusted the *p*‐values to control the False Discovery Rate (FDR) at α = 0.05. All statistical analyses were performed using Python (3.11), utilizing the SciPy (1.14.1) and Statsmodels (0.14.3) libraries.

#### 2.4.2 Workflow efficiency

The legacy TLD‐based process utilized a Harshaw 3500 system, which required a sequence of manual interventions. Each TLD‐100 capsule had to be individually cut open, the TLD power was transferred onto the reader (heating) platform, and the thermal heating/readout initiated. Efficiency for this cohort was defined as the total readout time for all 40 TLD capsules.

For film‐based IVD, multiple IVD segments (typically 12 to 16) were arranged within the central 5 × 10 cm^2^ region of the scanner bed to minimize LRAs. This arrangement allowed 40 IVD segments to be digitized in only 3–4 TIFF images for one orientation. These TIFF were subsequently imported in our data‐analysis platform to automated ROI detection and dose extraction. The workflow efficiency of the film system was evaluated as the cumulative time required for dual‐orientation scanning and automated software analysis.

## 3 RESULTS

Between October 2020 to August 2025, 11 patients diagnosed with MF underwent TSET at our institution. The TLD‐based IVD was used for 6 patients treated between October 2020 and September 2023 and the newly developed film‐based IVD was used for 5 patients treated from March 2024 and August 2025.

### 3.1 TLD‐based IVD

**FIGURE 2:**
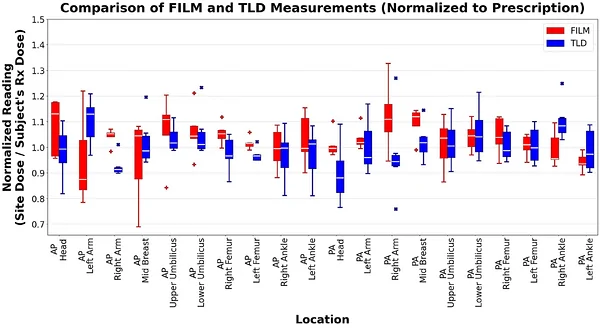
Comparison of in‐vivo dose measurements obtained using TLDs and EBT4 films. Boxplots display the distribution of readings normalized to the prescription dose, stratified by anatomical location. Red indicates film measurements, while blue indicates TLD measurements.

For the 6 TSET treatments monitored using the traditional TLD‐based system, the average dose of all patients and anatomical sites was 100.4% of the prescribed fractional dose, with a standard deviation (SD) of 9.7% (see Figure 2 for more details). This indicates high overall agreement with the prescription, though the standard deviation reflects the expected variability inherent in surface dosimetry for TSET.

As shown in Table 1, the Mann‐Whitney U test—supplemented by the Benjamini‐Hochberg correction for multiple comparison—confirmed that there were no significant differences in the delivered dose (*p* > 0.05) across any of the 20 monitored anatomical sites.

**TABLE 1:** Statistical significance of normalized dose (to prescription dose) differences at each site within the TLD cohort.

Site	Raw *P*‐Value	Number of TLD packets	Number of other TLD packets	Adjusted *P*‐Value (FDR)	Significant (alpha = 0.05)
AP Head	0.72	6	113	0.88	FALSE
AP Left Arm	0.02	6	113	0.11	FALSE
AP Right Arm	0.01	6	113	0.10	FALSE
AP Mid Breast	0.85	6	113	0.93	FALSE
AP Upper Umbilicus	0.27	6	113	0.68	FALSE
AP Lower Umbilicus	0.27	6	113	0.68	FALSE
AP Right Femur	0.52	6	113	0.88	FALSE
AP Left Femur	0.41	5	114	0.88	FALSE
AP Right Ankle	0.52	6	113	0.88	FALSE
AP Left Ankle	0.64	6	113	0.88	FALSE
PA Head	0.02	6	113	0.11	FALSE
PA Left Arm	0.74	6	113	0.88	FALSE
PA Right Arm	0.19	6	113	0.67	FALSE
PA Mid Breast	0.56	6	113	0.88	FALSE
PA Upper Umbilicus	0.75	6	113	0.88	FALSE
PA Lower Umbilicus	0.20	6	113	0.67	FALSE
PA Right Femur	0.89	6	113	0.93	FALSE
PA Left Femur	0.93	6	113	0.93	FALSE
PA Right Ankle	0.01	6	113	0.10	FALSE
PA Left Ankle	0.61	6	113	0.88	FALSE

### 3.2 Film‐based IVD

**FIGURE 3:**
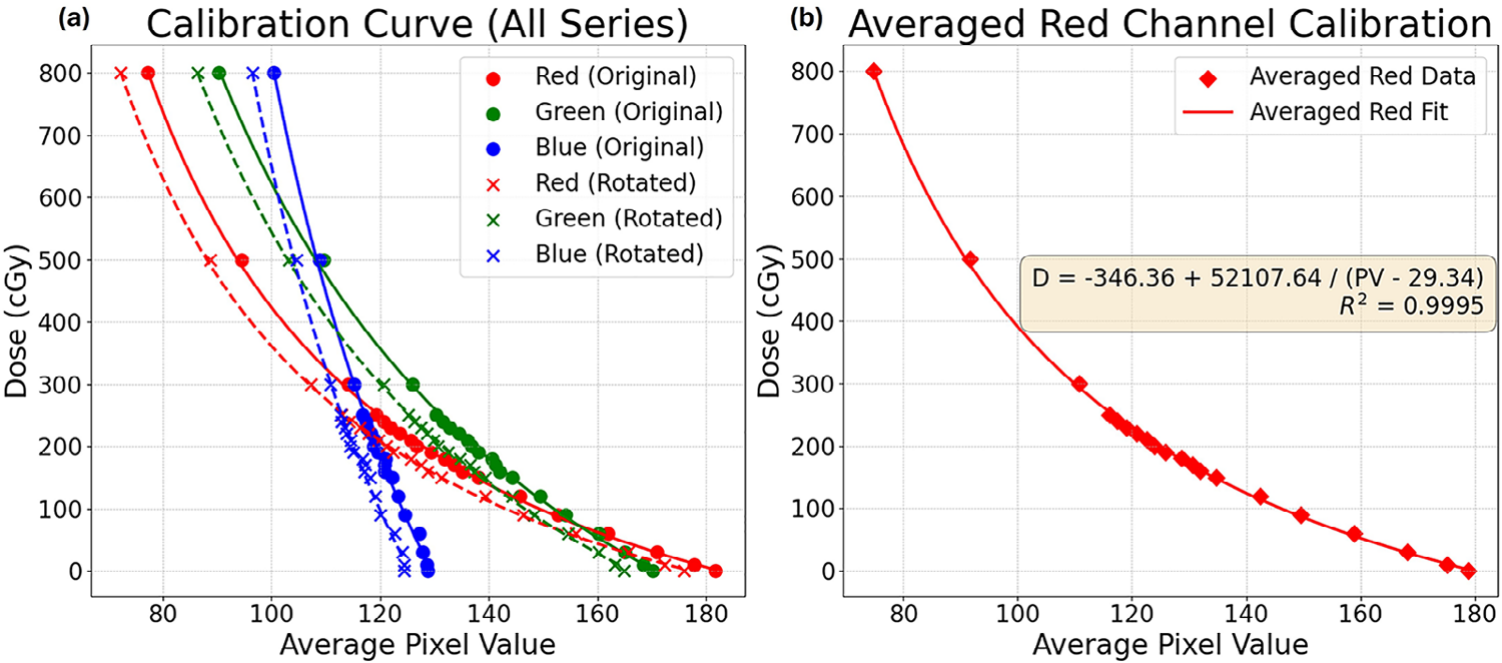
Radiochromic film calibration curves. (a) Dose‐response curves plotted for the red, green, and blue color channels across two film orientations (original and rotated). (b) Final calibration curve derived from the average red channel pixel values of both orientations, including the fitted rational function and correlation coefficient (R^2^).

Dose‐response calibration curves were constructed independently for the red, green, and blue channels. As shown in Figure 3, the red‐channel curve exhibited the widest dynamic range within the clinical dose range of interest (e.g., 200 cGy). In this region, the red channel demonstrated the highest signal‐to‐noise ratio, where fluctuations in PVs resulted in the smallest corresponding deviations in the calculated absorbed dose compared to the green or blue channels. Therefore, the red channel was selected for dose extraction in our analysis platform.

As shown in Figure 3, a notable separation exists between the red‐channel calibration curves of the original film and the 90°‐rotated film near the prescription dose levels. At a given PV, the relative difference in calculated dose reaches approximately 10%. To mitigate this orientation dependence, we implemented the orthogonal dual‐scan protocol, using the average red‐channel PV from both scans to establish a robust single calibration curve.

The relationship between the average red‐channel PV ($PV_{ave,\;red}$) and the absorbed dose was established using the following rational function:

$$D=-346.36+\frac{52107.64}{PV_{ave,\;red}-29.34}$$

This fitting function demonstrated an excellent correlation, with a coefficient determination of $R^{2}=0.9995$, indicating a robust and reliable relationship for converting orientation‐independent PVs into absolute absorbed dose.

The film‐based IVD system was clinically validated in five TSET treatments. The prescribed fractional dose was 200 cGy for four patients and 300 cGy for one patient. When normalized to the prescribed fractional dose, the average measured dose cross all patients and sites was 102.7% (SD = 9.6%; see Figure 2 for more details).

Statistical analysis using the Mann‐Whitney U test with Benjamini‐Hochberg correction showed no significant difference in delivered doses across the 20 monitored anatomical sites (*p* > 0.05; see Table 2).

**TABLE 2:** Statistical significance of normalized dose (to prescription dose) differences at each site within the film cohort.

Site	Raw *P*‐Value	Number of film packets	Number of other film packets	Adjusted *P*‐Value (FDR)	Significant (alpha = 0.05)
AP Head	0.26	5	95	0.75	FALSE
AP Left Arm	0.14	5	95	0.70	FALSE
AP Right Arm	0.66	5	95	0.81	FALSE
AP Mid Breast	0.51	5	95	0.81	FALSE
AP Upper Umbilicus	0.24	5	95	0.75	FALSE
AP Lower Umbilicus	0.55	5	95	0.81	FALSE
AP Right Femur	0.46	5	95	0.81	FALSE
AP Left Femur	0.69	5	95	0.81	FALSE
AP Right Ankle	0.44	5	95	0.81	FALSE
AP Left Ankle	0.98	5	95	0.98	FALSE
PA Head	0.51	5	95	0.81	FALSE
PA Left Arm	0.93	5	95	0.98	FALSE
PA Right Arm	0.10	5	95	0.65	FALSE
PA Mid Breast	0.05	5	95	0.52	FALSE
PA Upper Umbilicus	0.85	5	95	0.95	FALSE
PA Lower Umbilicus	0.69	5	95	0.81	FALSE
PA Right Femur	0.69	5	95	0.81	FALSE
PA Left Femur	0.48	5	95	0.81	FALSE
PA Right Ankle	0.26	5	95	0.75	FALSE
PA Left Ankle	0.01	5	95	0.15	FALSE

### 3.3 Comparison of TLD‐ and film‐based IVD

#### 3.3.1 Dose variability

The two IVD methods yielded high comparable dose statistics: the fractional dose for the film cohort was 102.7% ± 9.6%, compared to 100.4% ± 9.7% for the TLD cohort (see Figure 2).

A comprehensive analysis of the pooled measurement data (100 film vs. 119 TLD data points; one TLD data point was missing due to mishandling) showed no significant difference in dosimetric performance between the two IVD methods (*p* = 0.25). The finding confirms that the EBT4 film‐based system maintains dosimetric accuracy as the TLD‐based system (see Table 3).

**TABLE 3:** Statistical significance of differences in measured dose (normalized to prescription dose) between film and TLD cohort by anatomic locations.

Site	Raw *P*‐Value	Number of films	Number of TLDs	Adjusted *P*‐Value (FDR)	Significant (alpha = 0.05)
AP Head	0.13	5	6	0.36	FALSE
AP Left Arm	0.25	5	6	0.55	FALSE
AP Right Arm	0.01	5	6	0.17	FALSE
AP Mid Breast	0.93	5	6	1.00	FALSE
AP Upper Umbilicus	0.33	5	6	0.60	FALSE
AP Lower Umbilicus	0.66	5	6	0.88	FALSE
AP Right Femur	0.05	5	6	0.26	FALSE
AP Left Femur	0.10	5	5	0.32	FALSE
AP Right Ankle	0.93	5	6	1.00	FALSE
AP Left Ankle	0.66	5	6	0.88	FALSE
PA Head	0.05	5	6	0.26	FALSE
PA Left Arm	0.33	5	6	0.60	FALSE
PA Right Arm	0.08	5	6	0.32	FALSE
PA Mid Breast	0.25	5	6	0.55	FALSE
PA Upper Umbilicus	1.00	5	6	1.00	FALSE
PA Lower Umbilicus	1.00	5	6	1.00	FALSE
PA Right Femur	0.43	5	6	0.71	FALSE
PA Left Femur	0.93	5	6	1.00	FALSE
PA Right Ankle	0.05	5	6	0.26	FALSE
PA Left Ankle	0.66	5	6	0.88	FALSE

**FIGURE 4:**
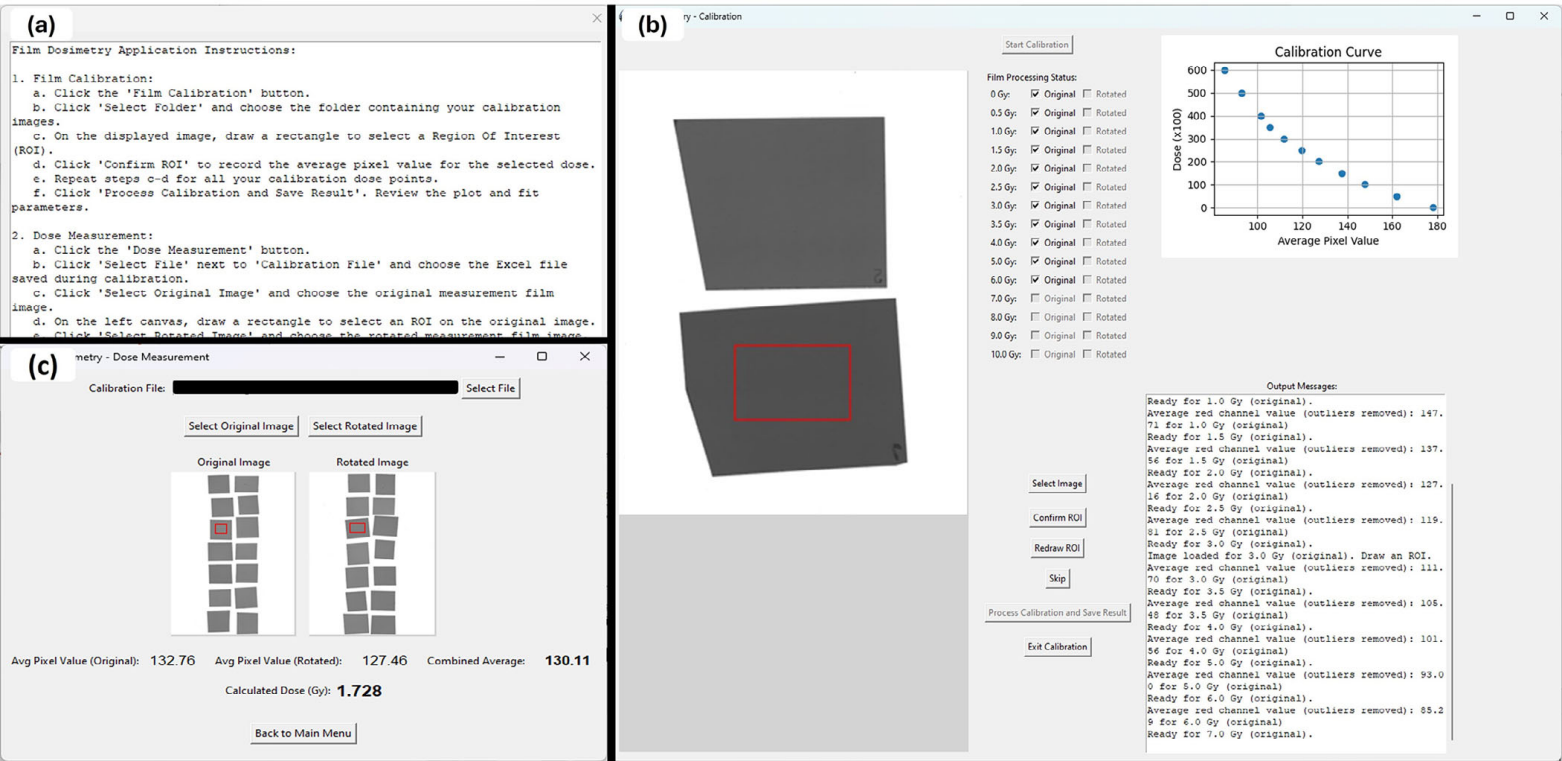
Graphical User Interface (GUI) of the Film Dosimetry program. (a) Operational instructions detailing the application workflow. (b) The calibration interface, featuring step‐by‐step guidance, region‐of‐interest (ROI) selection, and real‐time generation of the calibration curve. (c) The dose measurement interface used to calculate the delivered dose using the generated calibration file.

These results confirm that the EBT4‐based system, supported by the orthogonal dual‐scan protocol, verifies dose uniformity across the complex topography of the patient's surface, performing with clinical accuracy equivalent to the traditional TLD standard. The graphical‐user‐interface (GUI) of our data‐analysis software, including film calibration and film analysis, is shown in Figure 4.

#### 3.3.2 Workflow efficiency

On average, the end‐to‐end processing time of a single TLD capsule exceeded one min. For a complete TSET IVD session involving 40 capsules (two per anatomic site), the total readout time was approximately 50 min.

By utilizing the batch‐scanning capability of the flatbed scanner, all 40 IVD film segments were digitized in only 6–8 TIFF images (following the orthogonal dual‐scan protocol). The manual task of positioning a batch of films on the scanner bed and scanning them required approximately one min and a half. Once digitized, the images were imported into the data‐analysis platform for automated ROI detection and dose extraction. The total time required for the entire data extraction process was approximately 15 min per patient, saving approximately 35 min of clinical labor per TSET session.

## 4 DISCUSSIONS

Historically, our department has used TLD‐based in‐vivo dosimetry for TSET since early 2000s. However, due to high dosimetric variability, outdated equipment, and labor‐intensive readout process necessitate the evaluation of an improved alternative. In late 2023, we began developing a film‐based system, choosing the latest generation of Gafchromic™ EBT4 films due to the potential discontinuation of EBT3 films. While existing literature discusses film‐based IVD for TSET, most studies utilize older EBT2 or EBT3 films and rely on proprietary commercial software.^11^, ^13^, ^14^, ^15^, ^17^, ^18^

A primary challenge in film‐based IVD is the orientation‐dependent response of the active layer. When film is cut into small segments for clinical IVD using a standard guillotine cutter, maintaining a consistent notch for orientation is impractical and often results in imperfectly square segments. Our method overcomes this by utilizing an orthogonal dual‐scan protocol. By averaging the PVs from two perpendicular scans, the system becomes largely orientation independent. In addition, our film‐based IVD workflow reduced the readout time to about 15 min, compared to 50 min in TLD‐based IVD workflow.

Consistent with the findings in AAPM Task Group 235 and recent publications, we observed that EBT4 films exhibit a time‐dependent response due to post‐irradiation polymerization.^16^, ^19^, ^20^ While it is possible to model this change, we opted to standardize the readout at 24 h after the second day of irradiation, thus maintaining consistency with the standard waiting period used for TLD readouts.

Film‐based dosimetry was commissioned and validated using ion chambers, TLDs, and Metal‐Oxide‐Semiconductor Field‐Effect Transistors (MOSFETs). Overall, film measurements demonstrated good agreement with those from the other detectors, well within acceptable clinical tolerance limits. To ensure the robustness of our film‐based IVD system, we implemented a rigorous pre‐treatment QA using the abdominal section of an anthropomorphic RANDO phantom and a TBI spoiler. For the spot check, four dosimeter packets were taped to the RANDO phantom's surface at the cardinal angles (0°, 90°, 180°, and 270°). The phantom was placed on the TBI stand and aligned such that the umbilicus was positioned at the level of the gantry crosshair at the 270° projection, corresponding to the treatment isocenter. During this validation, the phantom was rotated to six standard treatment positions to deliver a full 12‐field irradiation, simulating a complete treatment fraction. Furthermore, the TBI spoiler was utilized to verify beam uniformity vertically and horizontally. Four additional dosimeter packets were placed on the TBI spoiler at positions approximately 60 cm superior and inferior to the isocenter, and 25 cm lateral to the spoiler midline. With the TBI spoiler set to an SSD of 350 cm, two angled TSET fields were delivered. All dosimeters, whether films or TLDs, were processed 24 h post‐irradiation to account for signal stability. In all validation cases, the dosimetric results met institutional requirements for output constancy and uniformity.

These phantom checks were supplemented by daily and monthly checks for output constancy, flatness and symmetry of the 6 MeV HD electron beam.

We utilized a 6 MV photon beam for calibration rather than a 6 MeV electron beam. This decision was supported by a recent study demonstrating that EBT4 films exhibit minimal energy dependence between megavoltage photon and electron beams.^16^ A significant advantage of this approach is that a single, robust photon‐based calibration curve can be applied to diverse clinical applications, including volumetric‐modulated arc therapy (VMAT)‐TBI and conventional electron therapy, simplifying the dosimetry workflow across the entire department.

TSET is an infrequently performed treatment at our institution, with 2–3 patients treated per year over the past five years. Therefore, only 11 patients are available during the study period.

During preparation of this manuscript, we noted that Ashland, the manufacture of Gafchromic™ EBT4 film, also offers a commercial product (pnt‐dos™ device) for in vivo dosimetry.

## 5 CONCLUSIONS

An in‐vivo dosimetry system using Gafchromic™ EBT4 film was successfully implemented for total skin electron therapy, providing a high‐performance alternative to traditional TLD‐based methods. Our findings demonstrate that the EBT4 film‐based approach improves workflow efficiency by 70%, reducing the total readout and analysis time from approximately 50 min to 15 min per patient.

Critically, this efficiency was achieved without compromising dosimetric accuracy; the film‐based system demonstrated clinical equivalence to the TLD‐based system (*p *= 0.25) while maintaining a compared standard deviation (9.6% vs. 9.7%). The integration of Gafchromic™ EBT‐4 film with a custom, open‐source Python analysis platform provides a robust, efficient, and cost‐effective solution for TSET IVD. Furthermore, the developed orthogonal dual‐scan protocol successfully mitigates the orientation‐dependence of IVD films, offering a universal solution for high‐throughput in‐vivo dosimetry in other radiotherapy applicators.

## AUTHOR CONTRIBUTIONS

**Zhexuan Zhang**: Conceptualization; methodology; software; analysis; writing — editing and review. **Lan Lu**: Conceptualization; methodology; writing — editing and review. **Gregory Videtic**: writing — review. **Sheen Cherian**: writing — review. **Ping Xia**: Supervision; writing — review. **Peng Qi**: Conceptualization; methodology; software; writing — editing and review; supervision.

## CONFLICT OF INTEREST STATEMENT

The author declares no conflicts of interest

## Data Availability

The data/code that support the findings of this study are available from the provided GitHub page in the article.

## References

[1] Bradford PT , Devesa SS , Anderson WF , et al. Cutaneous lymphoma incidence patterns in the United States: a population‐based study of 3884 cases. Blood. 2009;113:5064–5073. 19279331 10.1182/blood-2008-10-184168 PMC2686177

[2] Olsen EA , Whittaker S , Kim YH , et al. Clinical end points and response criteria in mycosis fungoides and Sezary syndrome: a consensus statement of the international society for cutaneous lymphomas, the United States cutaneous lymphoma consortium, and the cutaneous lymphoma task force of the European organisation for research and treatment of cancer. J Clin Oncol. 2011;29:2598–2607. 21576639 10.1200/JCO.2010.32.0630 PMC3422534

[3] Foss FM , Girardi M . Mycosis Fungoides and Sezary Syndrome. Hematol Oncol Clin North Am. 2017;31:297–315. 28340880 10.1016/j.hoc.2016.11.008

[4] Vaidya T , Badri T . Mycosis Fungoides. StatPearls. Treasure Island (FL); 2025.

[5] Karzmark CJ , Anderson J , F S , “Total Skin Electron Therapy: Technique and Dosimetry AAPM Report No. 23,” 1987. doi:10.37206/22

[6] GMM V , Vassil AD , Woody NM . Handbook of Treatment Planning in Radiation Oncology. 3rd ed. Springer Publishing Company; 2020.

[7] Specht L , Dabaja B , Illidge T , et al. Modern radiation therapy for primary cutaneous lymphomas: field and dose guidelines from the international lymphoma radiation oncology group. Int J Radiat Oncol Biol Phys. 2015;92:32–39. 25863751 10.1016/j.ijrobp.2015.01.008

[8] Specht L . Total skin electron beam therapy. Front Oncol. 2025;15:1498855. 40236646 10.3389/fonc.2025.1498855 PMC11997445

[9] Morris S , Scarisbrick J , Frew J , et al. The results of low‐dose total skin electron beam radiation therapy (TSEB) in patients with mycosis fungoides from the UK cutaneous lymphoma group. Int J Radiat Oncol Biol Phys. 2017;99:627–633. 28843374 10.1016/j.ijrobp.2017.05.052

[10] Dhivya S , Anuradha C , Murali V , et al. In‐vivo dosimetry in total skin electron therapy: literature review. J Radiother Pract. 2020;20:357–360.

[11] Bufacchi A , Carosi A , Adorante N , et al. In vivo EBT radiochromic film dosimetry of electron beam for total skin electron therapy (TSET). Phys Med. 2007;23:67–72. 17568545 10.1016/j.ejmp.2007.03.003

[12] Lewis D , Micke A , Yu X , et al. An efficient protocol for radiochromic film dosimetry combining calibration and measurement in a single scan. Med Phys. 2012;39:6339–6350. 23039670 10.1118/1.4754797 PMC9381144

[13] Falahati L , Nedaie HA , Esfahani M , et al. Dosimetric evaluation of electron total skin irradiation using gafchromic film and thermoluminescent dosimetry. J Cancer Res Ther. 2019;15:S115–S122. 30900632 10.4103/jcrt.JCRT_1020_16

[14] Baba MH , Singh BK , Wani SQ . In vivo dosimetry for dose verification of total skin electron beam therapy using gafchromic(R) EBT3 film dosimetry. J Med Phys. 2022;47:362–366. 36908494 10.4103/jmp.jmp_72_22 PMC9997533

[15] Sundaramoorthy D , Chandrasekaran A . Patient dose analysis using gafchromicTM EBT3 film: a retrospective study with a four dual‐field technique in total skin electron therapy. Asian Pac J Cancer Prev. 2023;24:2505–2513. 37505785 10.31557/APJCP.2023.24.7.2505 PMC10676495

[16] Guan F , Chen H , Draeger E , et al. Characterization of gafchromic(TM) EBT4 film with clinical kV/MV photons and MeV electrons. Precis Radiat Oncol. 2023;7:84–91. 40337264 10.1002/pro6.1204 PMC11935191

[17] Schiapparelli P , Zefiro D , Massone F , et al. Total skin electron therapy (TSET): a reimplementation using radiochromic films and IAEA TRS‐398 code of practice. Med Phys. 2010;37:3510–3517. 20831057 10.1118/1.3442301

[18] Cappelletto NIC , Shim MG . Total skin electron therapy vertical profiles measured using radiochromic film. J Appl Clin Med Phys. 2023;24:e13941. 36812051 10.1002/acm2.13941 PMC10161074

[19] Draeger E , Guan F , Lee MY , et al. Clinical use of Gafchromic EBT4 film for in vivo dosimetry for total body irradiation. J Appl Clin Med Phys. 2025;26:e14574. 39611814 10.1002/acm2.14574 PMC11905243

[20] Niroomand‐Rad A , Chiu‐Tsao ST , Grams MP , et al. Report of AAPM task group 235 radiochromic film dosimetry: an update to TG‐55. Med Phys. 2020;47:5986–6025. 32990328 10.1002/mp.14497

